# Trends, Coverage and Influencing Determinants of Influenza Vaccination in the Elderly: A Population-Based National Survey in Spain (2006–2017)

**DOI:** 10.3390/vaccines8020327

**Published:** 2020-06-19

**Authors:** Silvia Portero de la Cruz, Jesús Cebrino

**Affiliations:** 1 Department of Nursing, Pharmacology and Physiotherapy, Faculty of Medicine and Nursing, University of Córdoba, Avda. Menéndez Pidal, S/N, 14071 Córdoba, Spain; 2 Department of Preventive Medicine and Public Health, Faculty of Medicine, University of Seville, Avda. Doctor Fedriani, S/N, 41009 Seville, Spain; jcebrino@us.es

**Keywords:** aged, influenza, human, influenza vaccines, vaccination coverage, trends

## Abstract

Influenza is a significant public health problem and the elderly are at a greater risk of contracting the disease. The vaccination coverage of the elderly is below the Spanish target of 65% for each influenza season. The aims of this study were to report the coverage of influenza vaccination in Spain among the population aged ≥65 years and high-risk groups for suffering chronic diseases, to analyze the time trends from 2006 to 2017 and to identify the factors which affect vaccination coverage. A nationwide cross-sectional study was conducted including 20,753 non-institutionalized individuals aged ≥65 years who had participated in the Spanish National Health Surveys in 2006, 2011/2012, and 2017. Sociodemographic, health-related variables, and influenza vaccination data were used. A logistic regression analysis was performed to determine the variables associated with influenza vaccination. Influenza vaccination coverage was 60%. By chronic condition, older people with high cholesterol levels and cancer had the lowest vaccination coverage (62.41% and 60.73%, respectively). This coverage declined from 2006 to 2017 in both groups. Higher influenza vaccination was associated with males, Spanish nationality, normal social support perceived, polypharmacy, worse perceived health, participation in other preventive measures, and increasing age and the number of chronic diseases.

## 1. Introduction

Influenza is a significant public health problem which causes high mortality and morbidity rates [1]. Globally, seasonal influenza epidemics are estimated to lead to between 3 and 5 million cases of severe illness, while up to 650,000 persons die as a result [2], with most related deaths occurring among the elderly population and those with underlying chronic diseases [3]. Influenza also generates high social and health care costs [4,5].

Nowadays, the annual influenza vaccine is considered the main and most effective strategy to reduce the mortality and morbidity resulting from influenza, and its associated complications in high-risk individuals [6,7,8,9]. In Europe, the seasonal influenza vaccine is recommended for the elderly population and those at increased risk of influenza complications and severe disease, as well as for patients with chronic conditions [10]. Even though vaccination against influenza is free in Spain in all these cases, and that patients are normally vaccinated in primary care health centers [11], the vaccination coverage reported from representative national surveys is below 60% in older people and continues to be lower than the desired rate [12,13]. Here, the World Health Organization (WHO) recommends a minimum 75% vaccination coverage for the elderly population; however, in Spain, the objective is to reach 65% coverage in this group and to move closer to the goal established by the WHO [14]. The impact of not reaching an optimal vaccination uptake on public health is relevant, since a 1% decrease in associate mortality has been estimated to result from a 2.2% absolute increase in vaccination coverage [15].

In the European countries in 2017, 44.3% of people aged 65 and over were vaccinated against influenza [16] and 67.5% in the United States in 2016 [17]. Nowadays, people aged ≥65 years currently constitute 19.40% of the Spanish population [18]. In addition, the Spanish National Statistics Office predicts that this proportion will increase to 25.20% by 2033 [19]. Due to these situations as well as the lack of favorable evolution of the vaccination coverage, there is a clear need to study the factors related to regular vaccination. In that sense, prompt and efficient strategies to overcome barriers to vaccination could be devised. The main objectives of the present study were to report the coverage of influenza vaccination in Spain among the population aged ≥65 years and high-risk groups for suffering chronic medical conditions, to analyze time trends from 2006 to 2017, and to identify vaccination uptake factors.

## 2. Materials and Methods

### 2.1. Design and Study Population

A nationwide cross-sectional descriptive study was carried out from February to April 2020 using the data obtained from the records of the Spanish National Health Survey (SNHS) 2006 (from June 2006 to June 2007) [20], 2011/2012 (from July 2011 to June 2012) [21], and 2017 (from October 2016 to October 2017) [22]. The SNHS is a representative survey of the general population carried out by the Ministry of Health, Consumer Affairs and Social Welfare in partnership with the National Institute of Statistics, whose representativeness is ensured by assigning a weighting coefficient to each participant. This survey is conducted in non-institutionalized Spanish residents through personalized interviews. The sampling design was multistage probabilistic, stratified by census areas (first stage), family homes (second stage), and individuals (third stage). The present study included people aged ≥65 years old resident in Spain. Adults with dual nationality were excluded from the study. The initial sample consisted of 20,753 subjects (7835 in 2006; 5896 in 2011/2012; and 7022 in 2017), but, due to a lack of data for some of the variables studied, 5446 (26.24%) were excluded when the multivariate statistical analyses were carried out. The excluded subjects did not differ systematically from the rest of sample.

### 2.2. Variables

The dependent variable was “influenza vaccination”. This variable was assessed through the question: “Were you vaccinated against influenza during the last vaccination campaign?” Participants who answered “yes” were considered as having been vaccinated.

The independent variables were as follows.

Sociodemographic variables were: Year of survey (2006, 2011/2012, 2017); gender (male, female); age (65–74 years, 75–84 years, ≥85 years); nationality (Spanish, foreigner); level of education (without studies, primary, secondary or professional training, university); marital status (single, married, widowed, separated/divorced); size of town of residence (<10,000 inhabitants, 10,000–100,000 inhabitants, >100,000 inhabitants); and social class, which was assigned according to the categories proposed by the Spanish Society of Epidemiology [23] and classified as Class I (directors and managers of companies with 10 or more employees and professionals normally qualified with university degrees), Class II (directors and managers of companies with fewer than 10 salaried employees, professionals normally qualified with university degrees and other technical support professionals, athletes, and artists), Class III (intermediate professions and self-employed workers), Class IV (supervisors and workers in skilled technical work), Class V (skilled workers in the primary sector and other semi-skilled workers), and Class VI (unskilled workers). In this study, these six original classes were reduced to three groups from lowest to highest socioeconomic status (Classes I and II, Classes III and IV, and Classes V and VI).

Health-related variables were: Self-perceived health (very good, good, fair, poor, very poor); blood cholesterol level measurement in the last year (yes, no); blood pressure taken by a healthcare professional in the last year (yes, no); degree of limitation due to a health problem for at least six months (severely limited, limited but not severely, not at all limited); and body mass index (BMI), which was calculated from the self-reported body weight and height and categorized according to the World Health Organization [24] as underweight (BMI <18.50 kg/m^2^), normal-weight (BMI 18.50–24.99 kg/m^2^), overweight (BMI 25.00–29.99 kg/m^2^), and obese (BMI ≥30 kg/m^2^). To identify those chronic conditions for which annual influenza vaccination was recommended in Spain, the participants were classified as having the disease if they answered affirmatively to the question “Have you ever been diagnosed by a physician with any of the following diseases?” after showing them a list including diabetes, cancer, asthma, chronic bronchitis/emphysema/chronic obstructive pulmonary disease, arterial hypertension, myocardial infarction, cholesterol, and ictus; presence of chronic conditions (none, 1–2, ≥3); and polypharmacy, which was calculated using an identical question in all the questionnaires “From the following list of types of medication, which have you consumed in the last two weeks?”. Subjects were classified as polypharmacy if they gave an affirmative answer to the question for ≥5 different medication groups (including those used to treat diseases such as colds, flu, throat or lung infections, symptoms such as pain or fever, or laxatives). Although no consensus has yet been reached on the definition of polypharmacy, the threshold of ≥5 different medications was chosen because it has been used in recent studies conducted in different countries [25,26,27] and it is the most widely-used formula [28].

To measure qualitative and functional aspects of social support, the Duke-UNC-11 questionnaire [29], adapted to the Spanish population [30,31], was used. This scale includes 11 items which are scored on a Likert-like scale ranging from 1 (“Much less than I would like”) to 5 (“As much as I would like”). The overall perceived social support is obtained by adding the scores of the 11 items. The results range from 11 to 55 points. The final score is a dichotomized measure of the perceived social support: optimal support (≥33 points) and sup-optimal support (≤32 points).

### 2.3. Procedure

The data obtained from these surveys are available in the National Institute of Statistics and Ministry of Health, Consumer Affairs and Social Welfare websites [18,19,20] and were downloaded from these sites. According to the SNHS methodology, the microdata files are anonymous and are available to the public. In accordance with Spanish legislation, when secondary data are used, there is no need for approval from an Ethics Committee. The data research is available in the Supplementary File S1.

### 2.4. Data Analysis

A descriptive analysis was performed by calculating the counts and percentages for the qualitative variables and the quantitative variables by calculating the arithmetic mean and standard deviation (SD). The proportions of categorical variables were compared using the chi-square test for contingency tables or Fisher’s exact test if the number of expected frequencies was greater than 5. In addition, a logistic regression was performed to identify the variables associated with influenza vaccination. In the multivariate analysis, variables showing potential association with influenza vaccination (*p* ≤ 0.15) in the univariate analysis were included, and backward selection was used to eliminate non-significant variables based on the probability of the Wald statistic. Raw and adjusted odds ratios were calculated with 95% confidence intervals. The goodness of fit was verified with the Hosmer–Lemeshow test. All the hypothesis contrasts were bilateral and, in all the statistical tests, those with a 95% confidence level (*p* < 0.05) were considered significant values. The statistical analysis was carried out using IBM SPSS Statistics version 25 (IBM Corp, Armonk, NY, USA).

## 3. Results

### 3.1. Sociodemographic and Health-Related Variables

The total sample was 20,753 records of people ≥65 years old. The subjects had the following characteristics: predominantly female gender (62.01%; CI 95% 61.34–62.66%) and a mean age of 75.63 years (SD ± 7.22, CI 95% 75.53–75.72). Additionally, 38.11% had completed secondary or professional training (CI 95% 37.45–38.77%), 49.94% were married (CI 95% 49.26–50.62%), 42.92% belonged to social Classes V and VI (CI 95% 42.25–43.59%), 99.16% were Spanish (CI 95% 99.02–99.27%), and 39.17% lived in towns with a population of over 100,000 inhabitants (CI 95% 38.51–39.84%). In terms of body mass index, 37.30% were overweight (CI 95% 36.64–37.96%) (underweight (BMI <18.50 kg/m^2^), normal-weight (BMI 18.50–24.99 kg/m^2^), overweight (BMI 25.00–29.99 kg/m^2^), and obese (BMI ≥30 kg/m^2^)). On the other hand, 36.29% considered their health status good when answering a subjective question on the topic (CI 95% 35.64–36.95%). A total of 71.87% had, at least, three chronic conditions (CI 95% 71.25–72.48%), 75.81% had no polypharmacy (CI 95% 75.22–76.39%), and 55.70% did not report any degree of limitation in the last six months (CI 95% 55.03–56.38%). The social support was also assessed and 90.75% of participants perceived a normal level of social support (CI 95% 90.35–91.14%). Regarding the participation in other control and prevention measures against chronic conditions, the participation in influenza vaccination (60%; CI 95% 59.38–60.71%) was inferior to that for blood pressure (98.22%; CI 95% 98.03–98.39%) and cholesterol (95.31%; CI 95% 95.02–95.59%) measurements.

According to the year of the survey (Table 1), an increase in the number of older people with severe limitation due to a health problem (2006: 10.23%; 2011/2012: 9.75%; and 2017: 10.78%; *p* < 0.001) and belonging to the social Classes V and VI (2006: 29.74%; 2011/2012: 52.53%; and 2017: 49.56%; *p* < 0.001) can be seen.

### 3.2. Influenza Vaccination

**Figure 1:**
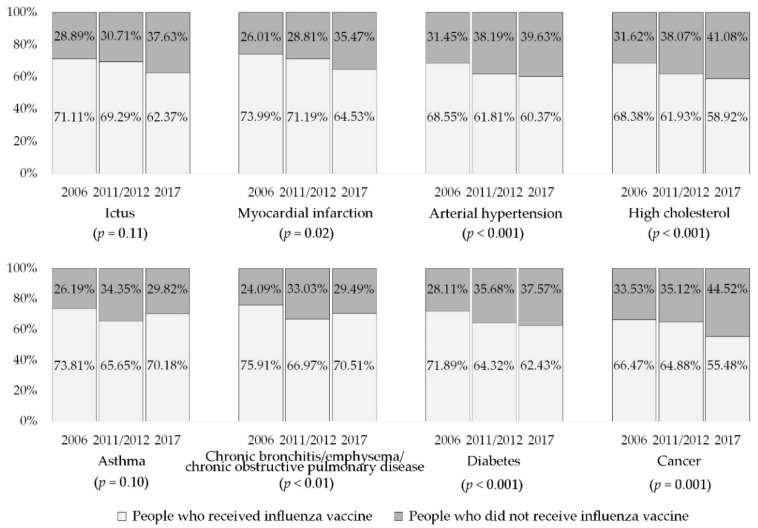
Patterns of influenza vaccination coverage according to chronic diseases in people aged ≥65 years in Spain. Spanish National Health Surveys 2006–2017.

The prevalence of influenza immunization decreased from 2006 to 2017 (2006: 66.08%; 2011/2012: 58.16%; and 2017: 54.91%; *p* < 0.001). As regards chronic conditions, we found the highest administration of vaccine among those older people with bronchitis, emphysema, and/or chronic obstructive pulmonary disease (71.40%), followed by asthma (70.52%), myocardial infarction (69.87%), and ictus (66.61%). The lowest figures are shown in those with diabetes (65.69%), arterial hypertension (63.52%), high cholesterol (62.41%), and cancer (60.73%). There were significant differences in the numbers receiving influenza vaccination in the different years of the study and the presence of chronic conditions. Over the years, the participation in influenza vaccination campaigns which decreased most was among older people with diabetes (2006: 71.89%; and 2017: 62.43%; *p* < 0.001), high cholesterol (2006: 68.38%; and 2017: 58.92%; *p* < 0.001), and myocardial infarction (2006: 73.99%; and 2017: 64.53%; *p* = 0.02) (Figure 1).

### 3.3. Association between Sociodemographic and Health-Related Variables and Influenza Vaccination

The influenza vaccination is distributed differently according to sociodemographic and health-related characteristics (Table 2). In the bivariate analysis, significant differences were observed for all the sociodemographic and health status variables between those older people who had been vaccinated against influenza and those who had not. In the multivariate analysis, the probability of people aged ≥65 years old participating in the influenza vaccination campaign was higher in males (of the total sample, 37.99% were males), in people who had participated in blood pressure and cholesterol testing campaigns, those who perceived normal social support, and those with polypharmacy, and was lower in foreign participants. Moreover, a clear upward trend was observed in this probability with increased age and number of chronic diseases, and when the perceived health was worse.

## 4. Discussion

The National Health Survey is an important tool that has been used in previous studies because of its statistical potential as a representative national survey with a considerable sample size. However, in the present study, foreign participants seem to be underrepresented. Both international [32] and national [33] studies show that the foreign population has a lower participation in population social and health surveys. Most of the identified barriers to participation in the health survey do not appear to be related to ethnic background alone but reflect general issues pertaining to questionnaires of this length and complexity. In addition, level of education and language barriers may be factors especially influencing response rate [34].

The results of the present study provide an update on the mean influenza vaccination coverage rate in older people in Spain, which is currently 60%. Although vaccination has been recommended for many years, a huge disparity exists between the vaccination coverage of different countries (Table 3).

The different vaccination rates between those countries might be explained by the different publicity campaigns supporting the vaccination recommendations, differences in the vaccination systems and funding schemes, and different attitudes related to seasonal influenza vaccination. Although there has been widespread consensus for many years that older age groups should be vaccinated, and the WHO urges Member States of the Europe Union to carry out interventions and strategies to facilitate increased influenza vaccination coverage for the elderly population, the United Kingdom was the only country to come near to the Europe Union target of 75% in the 2015/2016 and 2016/2017 influenza seasons [38]. In the present study, the influenza vaccination coverage ranged from 66.08% in 2006 to 54.94% in 2017. Despite the fact that, in some European countries (Germany, Sweden, Portugal, Luxembourg, Poland, Slovenia, Denmark, Ireland, Italy, France, Netherlands, Hungary, Romania, and Italy) an increasing trend was observed before 2009 pandemic [39], public discussion about the effectiveness, safety, and necessity of influenza vaccination, especially during the 2009 pandemic, has created doubt among the general public about getting vaccinated against influenza. This doubt may have contributed to the declining trend in influenza vaccination coverage in most European countries over the last few years [40,41]. Other factors that may be considered in decreasing coverage are conflicting messages about pandemic risk [42] and issues of complacency (lower perceived severity of disease, lower perceived risk of disease) [43,44].

When analyzing predictors of influenza vaccination uptake (Table 2), males were associated with higher rates of vaccination. Similar results have been reported in other studies from recent years [15] and these could be explained by women reporting more adverse reactions to vaccines or there being a lack of recommendations for them, as women are usually considered to be more aware of self-health care and prevention compliance habits than men; another explanation could be a decreased perception of vaccine safety and efficacy [45]. However, in other studies, either vaccination coverage was reported to be higher among females [46,47,48] or no associations were found between those variables [49]. Three studies similar to ours performed in several countries found that older people (>70 years) were associated with being more compliant regarding influenza vaccination [48,50,51]. Nevertheless, Ganczak et al. [52] revealed that younger patients (<70 years) were almost eight times more likely to receive the influenza vaccine, due to the fact that old age is often associated with imperfect functional status, which may negatively influence the likelihood of vaccine uptake, since access might depend on transportation or assistance. What these findings all show, however, is that further evidence is needed. Martínez et al. [37] found that foreigners were less likely to uptake the vaccine than Spanish people, in line with the results of the present study. Some prior studies have reported similar findings regarding social and racial disparities on vaccination [53,54,55]. This may reflect the lesser degree of primary medical care in the foreign population, who are subsequently less likely to have regular vaccinations [43,56]. Spain provides access to healthcare to foreign people on the same basis as to people with Spanish nationality in order that foreigners may not seek preventative healthcare services due to lacking awareness of entitlements [57].

In the univariate analysis, older people with a lower educational level were more likely to participate in influenza vaccination campaigns compared to those with university studies. This finding is similar to that found in other studies [12,58]. Moreover, several studies conducted in European countries where the vaccination of elderly persons is provided free-of-charge found that educational level was not a major determinant of vaccination [49,59]. Regarding marital status, we found that being widowed increased the decision to become immunized while being separated or divorced decreased it. Despite some authors [60,61,62] finding no relationship between those variables, marital status is considered an important predictor of healthcare utilization. In fact, marriage may influence health status not only through the support and protection that marriage offers, but also through a more efficient pattern of healthcare utilization. As for social class, there was a difference of the social class distribution between 2006 and subsequent surveys. This may be due to the economic crisis that affected Spain from 2008 to 2013. Between 2009 and 2013, the real gross domestic product fell by 5.2%, and the unemployment rate increased from 8.6% in 2007 to 25.7% in 2013 [63]. Accordingly, the data for the post-2008 period indicate a transformation of the social class in Spain with an increase in the number of people belonging to the lower social class and a pronounced loss of weight for skilled manual workers belonging to the middle social class. On the other side, there an increase of people who belonging to upper social class, which may be explained by the well-proven fact that higher quality jobs (those that require more human capital) offer better working conditions and more protection against the job destruction associated with economic crisis [64]. In the univariate analysis, older people from social Classes V and VI had a higher influenza vaccination coverage than those from higher classes. This situation is similar to that found in some studies [12,65,66] and may be due to people from higher social classes possibly being more susceptible to both anti-vaccination campaigns and the increased perception of the potential risks of vaccinations [65].

Regarding the size of town, we found that the participation in influenza vaccination campaigns by older people who lived in areas with <10,000 inhabitants was higher than others who lived in cities with >100,000 inhabitants. In rural settings, the healthcare professionals have closer relationships with their patients, making it easier for them to promote local health services and personally recommend campaigns of vaccinations; in addition, the information is more willingly accepted by patients [67].

In the current study, the univariate analysis showed that influenza coverage was higher in overweight and obese older people than in those with a normal weight. According to a recent systematic review and meta-analysis, people with obesity may be both more likely to perceive higher risk from vaccine-preventable diseases and more accepting of the benefits and safety of recommended vaccinations [68]. On the other hand, the results of this study are in line with other studies [51,69,70,71], which show that poor self-perceived health status is associated with receiving vaccinations, while a good self-perceived health status is the most common reason for the refusal to be vaccinated. This finding can be explained because, among older people, the likelihood is that having a chronic illness will influence their self-perceived health status. In addition, we observed that individuals affected by one or more chronic diseases had a greater likelihood of being vaccinated against influenza than those reporting non-chronic conditions. Previous studies conducted in Spain [53] and in other countries [46,72,73,74] reported analogous findings. Despite the mistrust which older people with chronic conditions have of vaccination and the fact that they often have a lower perceived risk of influenza than of other without chronic diseases, being advised to take the influenza vaccination by their doctor or nurse is a good way to promote vaccination uptake [46]. On the other hand, people without chronic diseases feel that influenza is not a dangerous disease and more often than chronic patients choose not to be vaccinated [46]. In addition, in the current study, patients with bronchitis, emphysema, and/or chronic obstructive pulmonary disease and those with asthma showed the highest vaccination coverage. This finding may be explained by the fact that respiratory diseases are closely related to influenza complications, such a pneumonia, and vaccination is strongly recommended and effective to prevent hospitalization among older people [75,76,77,78]. Regarding influenza vaccination as a recommended step for cancer patients, cancer survivors, and those who are not currently undergoing treatment [79], we observed that the lowest rate of influenza vaccination coverage was among the older people with cancer. In line with our findings, vaccination rates found among these population in other studies were also very low [74,78,80]. Another relevant result regarding individuals with chronic diseases was that the biggest decrease in vaccination coverage from 2006 to 2017 was identified in older people with diabetes, cholesterol, and myocardial infarction, which points to the need to conduct studies to understand the barriers or aspects of patient behavior which account for this result (Figure 1).

In this study, an overwhelming majority (>95%) had had their blood pressure and cholesterol measured in the last year and 59.15% had been given the influenza vaccination. In the multivariate analysis, clinical preventive practices such as taking blood pressure and cholesterol values increased the probability of participation in vaccination campaigns for influenza. In that sense, Spanish National Health System advises that any contact with the health system should be used to establish the influenza vaccination recommendation [81]. In another study using the same methodology [12], with an older population, 90.90% and 86.20% of the participants affirmed that less than one year had elapsed since they last had their blood pressure and blood cholesterol tested, respectively, and 63.36% had received influenza immunization. It seems clear that the elderly population with chronic conditions participate in other clinical preventive practices more than in influenza vaccination. This may occur because, for example, patients who suffer from arterial hypertension go to see their nurse to control their blood pressure while the reason for having an influenza vaccination is because older people are a risk population, and they do not always they agree with this. Taking into account that the cost-effectiveness results for influenza vaccination among adults is comparable to the cost-effectiveness of other preventive health interventions targeting adults, including colorectal screening, breast cancer screening, and arterial hypertension screening and treatment [82,83], healthcare professionals and healthcare systems should invest their time and efforts into promoting, implementing and incentivizing influenza vaccination programs to ensure all patients are given a strong recommendation for vaccination and have the opportunity to benefit from it. Furthermore, most studies point out that people with greater limitations are less likely to receive the recommended care for their chronic conditions. In fact, recommended care tends to decrease when the person’s limitations increase, and is substantially lower among severely limited patients [84], which hinders their adherence to vaccination campaigns.

Consistent with previous studies [85,86], our results show that older people with a normal social support were more likely to receive influenza vaccination than those with a lower social support. Social support is considered a predictor of a healthy general lifestyle [85]. Interaction with others has an impact on health-relevant behavior. They may support behavior change by offering instrumental help or emotional encouragement [87,88]. Recommendations and communication from healthcare personnel to improve the individual’s perceptions of their susceptibility to and the severity of a health threat are the major influences on the likelihood of that person behaving in a way to avoid the threat [89], which could facilitate vaccination uptake. This is an important issue to consider because vaccination may reduce the problems related to polypharmacy in the elderly population with many comorbidities, which may lead to major adverse effects or lack of compliance [90,91].

This study reflects the current state of influenza vaccination coverage in individuals aged 65 and older. It is vital therefore that our current knowledge about the factors that influence vaccine coverage should be taken into consideration by health authorities when designing strategies to improve influenza vaccination coverage. Even though there are several interventions which produce significant, positive effects, such as sending postcards (low intensity), making personalized phone calls (medium intensity), and making home visits (high intensity), all of which increase the community demand for vaccination, enhance access and improve the provider/system [92], it is not enough simply to identify the evidence about the benefits of a particular intervention. Those interventions require organized procedures, and systematic, integrated processes must be followed to achieve both its initial adoption and its medium- to long-term maintenance and convey to the elderly population the benefits provided by a cost-effective intervention such as the influenza vaccine. In this way, it would be possible to overcome the trend of decreasing influenza vaccination observed in Spain, which is falling to clearly insufficient vaccination coverage rates.

The present study also has some limitations. Firstly, due to the cross-sectional design, it is not possible to assign causality between vaccination and the related variables. It should be remembered that the data collected in the surveys was obtained indirectly from the informants’ self-reporting, which can be affected by memory and/or social desirability bias. Furthermore, the surveys also failed to ask the people why they had or had not been vaccinated, which would be interesting to include in future research. Moreover, the reliability of respondent’s answers about influenza vaccination cannot be verified. In addition, foreign participants seem to be underrepresented, so results obtained from these databases should be taken with caution. There are different strategies to increase the response rate in the foreign population. In that sense, it would be desirable to translate the questionnaire into various languages, to use surveyors belonging to the different communities, or to use specific types of sampling, such as “snowball”. On the other hand, one strength of our study is that since the data were derived from a national survey, they have been obtained using carefully planned methodology, including sampling, well-designed forms, preparation of the survey participants, supervision of the survey, and filtering of the data, all of which guarantee a representative sample of the population.

## 5. Conclusions

The coverage of influenza vaccination in the elderly population is 60%, which is clearly well below the recommended level. Older people with bronchitis, emphysema, and/or chronic obstructive pulmonary disease and those with asthma had the highest vaccination coverage and individuals with high cholesterol and cancer the lowest. There is a decreased participation in the influenza vaccination campaigns in older people, in general, and in elderly individuals with chronic conditions, in particular, from 2006 to 2017. The likelihood of being vaccinated for influenza is higher in males; in individuals who participate in other control and prevention measures against chronic conditions, such as blood pressure and cholesterol measurement campaigns; in those with normal perceived social support; and in those with polypharmacy. In addition, the older is the patient, the more chronic conditions they suffer from, and the worse their perceived health, the greater is the likelihood of them agreeing to be vaccinated. By contrast, this likelihood is lower in foreigners.

## Figures and Tables

**Table 1:** Sociodemographic and health-related characteristics of people aged ≥65 years in Spain. Spanish National Health Surveys 2006–2017.

Variables	2006	2011/2012	2017	*p*-Value
	*n* = 7835 (%)	*n* = 5896 (%)	*n* = 7022 (%)	
Gender				<0.001
Female	5022 (64.10)	3673 (62.30)	4173 (59.43)	
Male	2813 (35.90)	2223 (37.70)	2849 (40.57)	
Age group				<0.001
65–74 years	3931 (50.17)	2731 (46.32)	3382 (48.16)	
75–84 years	3157 (40.30)	2350 (39.86)	2543 (36.22)	
≥85 years	747 (9.53)	815 (13.82)	1097 (15.62)	
Nationality				<0.001
Spanish	7736 (98.74)	5826 (98.81)	7016 (99.91)	
Foreigner	71 (0.91)	69 (1.17)	2 (0.03)	
Do not know/answer	28 (0.35)	1 (0.02)	4 (0.06)	
Level of education				<0.001
University	522 (6.66)	325 (5.51)	634 (9.03)	
Secondary or PT	4388 (56.00)	1882 (31.92)	1638 (23.33)	
Primary	2428 (30.99)	1374 (23.30)	2614 (37.22)	
Without studies	473 (6.04)	2315 (39.27)	2136 (30.42)	
Do not know/answer	24 (0.31)	0 (0.00)	0 (0.00)	
Marital status				<0.001
Single	773 (9.87)	489 (8.29)	572 (8.14)	
Married	3886 (49.60)	2912 (49.39)	3566 (50.78)	
Widowed	2991 (38.17)	2310 (39.18)	2567 (36.56)	
Separated/Divorced	174 (2.22)	180 (3.05)	306 (4.36)	
Do not know/answer	11 (0.14)	5 (0.09)	11 (0.16)	
Social class				<0.001
Classes I and II	938 (11.97)	726 (12.31)	924 (13.16)	
Classes III and IV	4247 (54.21)	1690 (28.66)	2284 (32.53)	
Classes V and VI	2330 (29.74)	3097 (52.53)	3480 (49.56)	
Do not know/answer	320 (4.08)	383 (6.50)	334 (4.75)	
Size of town of residence				<0.001
>100,000 inhabitants	2810 (35.87)	2425 (41.13)	2894 (41.21)	
10,000–100,000 inhabitants	2379 (30.36)	1743 (29.56)	2260 (32.19)	
<10,000 inhabitants	2646 (33.77)	1728 (29.31)	1868 (26.60)	
Body Mass Index				<0.001
Underweight	64 (0.82)	47 (0.80)	72 (1.02)	
Normal weight	1738 (22.18)	1370 (23.23)	1978 (28.17)	
Overweight	2784 (35.53)	2116 (35.89)	2840 (40.44)	
Obese	1387 (17.70)	1158 (19.64)	1487 (21.18)	
Do not know/answer	1862 (23.77)	1205 (20.44)	645 (9.19)	
Self-perceived health				<0.001
Very good	430 (5.49)	359 (6.09)	441 (6.28)	
Good	2581 (32.94)	2205 (37.40)	2745 (39.09)	
Fair	3299 (42.11)	2175 (36.89)	2592 (36.92)	
Poor	1177 (15.02)	915 (15.52)	977 (13.91)	
Very poor	348 (4.44)	242 (4.10)	267 (3.80)	
Presence of chronic conditions				<0.001
None	394 (5.03)	360 (6.11)	347 (4.94)	
1–2	1842 (23.51)	1395 (23.66)	1500 (21.36)	
≥3	5599 (71.46)	4141 (70.23)	5175 (73.70)	
Blood cholesterol measurement				<0.001
No	379 (4.84)	317 (5.38)	49 (0.70)	
Yes	7259 (92.65)	5555 (94.21)	6966 (99.20)	
Do not know/answer	197 (2.51)	24 (0.41)	7 (0.10)	
Blood pressure measurement				<0.001
No	89 (1.14)	123 (2.09)	38 (0.54)	
Yes	7632 (97.41)	5770 (97.86)	6982 (99.43)	
Do not know/answer	114 (1.45)	3 (0.05)	2 (0.03)	
Degree of limitation due to a health problem for at least 6 months				<0.001
Not at all limited	4468 (57.03)	3562 (60.42)	3530 (50.27)	
Limited but not severely	2565 (32.74)	1757 (29.80)	2734 (38.94)	
Severely limited	802 (10.23)	575 (9.75)	757 (10.78)	
Do not know/answer	0 (0.00)	2 (0.03)	1 (0.01)	
Social support				<0.001
Low social support	347 (4.43)	251 (4.26)	400 (5.70)	
Normal social support	7040 (89.85)	5206 (88.30)	6588 (93.82)	
Do not know/answer	448 (5.72)	439 (7.44)	34 (0.48)	
Polypharmacy				<0.001
Yes	1570 (20.04)	1426 (24.19)	1901 (27.07)	
No	6208 (79.23)	4432 (75.17)	5093 (72.53)	
Do not know/answer	57 (0.73)	38 (0.64)	28 (0.40)	

PT, Professional Training; *p*-value is for trend.

**Table 2:** Association between participation in the influenza vaccination campaign, sociodemographic and health-related variables in people aged ≥65 years in Spain. Spanish National Health Surveys 2006–2017.

Variables	2006	2011/2012	2017	Total	OR (CI 95%)	*p*-Value	ORa (CI 95%)	*p*-Value
	*n* = 5177 (%)	*n* = 3429 (%)	*n* = 3856 (%)	*n* = 12,462 (%)				
Gender								
Female	3263 (63.03)	2122 (61.88)	2238 (58.04)	7623 (61.17)	Reference		Reference	
Male	1914 (36.97)	1307 (38.12)	1618 (41.96)	4839 (38.83)	1.09 (1.03–1.16)	<0.01	1.32 (1.23–1.41)	<0.001
Age group								
65–74 years	2312 (44.66)	1339 (39.05)	1520 (39.42)	5171 (41.49)	Reference		Reference	
75–84 years	2307 (44.56)	1541 (44.94)	1614 (41.86)	5462 (43.83)	1.99 (1.87–2.11)	<0.001	1.87 (1.74–2.02)	<0.001
≥85 years	558 (10.78)	549 (16.01)	722 (18.72)	1829 (14.68)	2.08 (1.90–2.27)	<0.001	2.08 (1.85–2.34)	<0.001
Nationality								
Spanish	5128 (99.05)	3399 (99.13)	3854 (99.95)	12,381 (99.35)	Reference			
Foreigner	30 (0.58)	30 (0.87)	0 (0.00)	60 (0.48)	0.42 (0.19–0.92)	<0.01	Reference	
Do not know/answer	19 (0.37)	0 (0.00)	2 (0.05)	21 (0.17)	0.86 (0.42–0.93)	<0.01	0.61 (0.41–0.90)	0.01
Level of education								
University	312 (6.03)	156 (4.55)	321 (8.33)	789 (6.33)	Reference			
Secondary or PT	2872 (55.48)	1056 (30.80)	800 (20.75)	4728 (37.94)	1.21 (1.14–1.48)	<0.001		
Primary	1645 (31.77)	831 (24.23)	1440 (37.34)	3916 (31.42)	1.11 (1.08–1.62)	<0.001		
Without studies	332 (6.41)	1386 (40.42)	1295 (33.58)	3013 (24.18)	1.13 (1.04–1.51)	<0.001		
Do not know/answer	16 (0.31)	0 (0.00)	0 (0.00)	16 (0.13)	1.27 (1.11–1.92)	<0.001		
Marital status								
Single	462 (8.92)	290 (8.46)	309 (8.01)	1061 (8.51)	Reference			
Married	2609 (50.40)	1680 (48.99)	1972 (51.14)	6261 (50.24)	1.12 (0.99–1.23)	0.06		
Widowed	2012 (38.86)	1385 (40.39)	1433 (37.17)	4830 (38.76)	1.16 (1.05–1.28)	0.01		
Separated/Divorced	90 (1.74)	71 (2.07)	140 (3.63)	301 (2.42)	0.61 (0.51–0.73)	<0.001		
Do not know/answer	4 (0.08)	3 (0.09)	2 (0.05)	9 (0.07)	0.36 (0.16–0.82)	0.01		
Social class								
Classes I and II	617 (11.92)	379 (11.05)	470 (12.19)	1466 (11.76)	Reference			
Classes III and IV	2830 (54.67)	994 (28.99)	1231 (31.92)	5055 (40.56)	1.22 (1.12–1.33)	<0.001		
Classes V and VI	1532 (29.59)	1840 (53.66)	1970 (51.09)	5342 (42.87)	1.15 (1.05–1.25)	0.01		
Do not know/answer	198 (3.82)	216 (6.30)	185 (4.80)	599 (4.81)	1.04 (0.91–1.21)	0.54		
Size of town of residence								
>100,000 inhabitants	1805 (34.87)	1379 (40.22)	1522 (39.47)	4706 (37.76)	Reference			
10,000–100,000 inhabitants	1530 (29.55)	992 (28.93)	1200 (31.12)	3722 (29.87)	1.02 (0.95–1.09)	0.6		
<10,000 inhabitants	1842 (35.58)	1058 (30.85)	1134 (29.41)	4034 (32.37)	1.33 (1.24–1.42)	<0.001		
Body Mass Index								
Underweight	41 (0.79)	25 (0.73)	34 (0.88)	100 (0.80)	Reference			
Normal weight	1111 (21.46)	767 (22.37)	1047 (27.15)	2925 (23.47)	0.82 (0.58–1.19)	0.16		
Overweight	1819 (35.14)	1228 (35.81)	1580 (40.98)	4627 (37.13)	1.11 (1.07–1.21)	0.01		
Obese	924 (17.85)	689 (20.09)	834 (21.63)	2447 (19.64)	1.13 (1.04–1.25)	0.01		
Do not know/answer	1282 (24.76)	720 (21.00)	361 (9.36)	2363 (18.96)	1.28 (0.95- 1.73)	0.1		
Self-perceived health								
Very good	227 (4.38)	153 (4.46)	169 (4.38)	549 (4.41)	Reference		Reference	
Good	1594 (30.79)	1170 (34.12)	1364 (35.37)	4128 (33.12)	1.51 (1.33–1.70)	<0.001	1.31 (1.14–1.52)	<0.001
Fair	2305 (44.52)	1371 (39.98)	1560 (40.46)	5236 (42.02)	2.30 (2.03–2.60)	<0.001	1.56 (1.35–1.81)	<0.001
Poor	812 (15.69)	591 (17.24)	585 (15.17)	1988 (15.95)	2.28 (1.99–2.61)	<0.001	1.37 (1.16–1.62)	<0.001
Very poor	239 (4.62)	144 (4.20)	178 (4.62)	561 (4.50)	2.35 (1.96–2.82)	<0.001	1.43 (1.13–1.80)	<0.01
Presence of chronic diseases								
None	159 (3.07)	140 (4.08)	99 (2.57)	398 (3.19)	Reference		Reference	
1–2	1058 (20.44)	672 (19.60)	661 (17.14)	2391 (19.19)	1.80 (1.57–2.06)	<0.001	1.57 (1.33–1.84)	<0.001
≥3	3960 (76.49)	2617 (76.32)	3096 (80.29)	9673 (77.62)	3.24 (2.87–3.70)	<0.001	2.31 (1.97–2.71)	<0.001
Blood cholesterol measurement								
No	186 (3.59)	126 (3.67)	17 (0.44)	329 (2.64)	Reference			
Yes	4887 (94.40)	3291 (95.98)	3839 (99.56)	12,017 (96.43)	1.96 (1.69–2.27)	<0.001	Reference	
Do not know/answer	104 (2.01)	12 (0.35)	0 (0.00)	116 (0.93)	1.31 (0.97–1.76)	0.08	1.33 (1.10–1.61)	<0.01
Blood pressure measurement								
No	34 (0.66)	36 (1.05)	10 (0.26)	80 (0.64)	Reference			
Yes	5093 (98.38)	3392 (98.92)	3846 (99.74)	12,331 (98.95)	3.25 (2.49–4.25)	<0.001	Reference	
Do not know/answer	50 (0.96)	1 (0.03)	0 (0.00)	51 (0.41)	1.59 (1.02–2.50)	0.04	1.70 (1.20–2.40)	<0.01
Degree of limitation due to a health problem for at least 6 months								
Not at all limited	2816 (54.40)	1945 (56.72)	1730 (44.87)	6491 (52.08)	Reference			
Limited but not severely	1778 (34.34)	1117 (32.58)	1642 (42.58)	4537 (36.41)	1.49 (1.32–1.78)	<0.001		
Severely limited	583 (11.26)	366 (10.67)	484 (12.55)	1433 (11.50)	1.44 (1.27–1.62)	<0.001		
Do not know/answer	0 (0.00)	1 (0.03)	0 (0.00)	1 (0.01)	1.50 (0.37–1.21)	0.1		
Social support								
Low social support	212 (4.09)	125 (3.65)	204 (5.29)	541 (4.34)	Reference			
Normal social support	4670 (90.21)	3043 (88.74)	3635 (94.27)	11,348 (91.06)	1.28 (1.13–1.46)	<0.001	Reference	
Do not know/answer	295 (5.70)	261 (7.61)	17 (0.44)	573 (4.60)	1.39 (1.16–1.67)	<0.001	1.41 (1.19–1.67)	<0.001
Polypharmacy								
No	3959 (76.47)	2440 (71.16)	2598 (67.38)	8997 (72.19)	Reference			
Yes	1181 (22.81)	963 (28.08)	1238 (32.10)	3382 (27.14)	1.67 (1.56–1.79)	<0.001	Reference	
Do not know/answer	37 (0.72)	26 (0.76)	20 (0.52)	83 (0.67)	1.55 (1.06–2.27)	0.02	1.32 (1.21–1.45)	< 0.001

PT, Professional Training; OR, odds ratio; ORa, odds ratio adjusted for all sociodemographic and health-related variables; CI 95%, 95% Confidence Interval; n, number of people participating in vaccination campaigns for influenza; Hosmer–Lemeshow test χ^2^ = 2.29, *p* = 0.94; Nagelkerke’s R^2^: 0.08; *p*-value < 0.001.

**Table 3:** Vaccination coverage of different countries.

Country	Year	Influenza Vaccination Coverage Rate in Older People
Netherlands [35]	2006	76%
	2011/2012	73.50%
	2013	68.80%
	2017	64%
Italy [35]	2006	68.35%
	2011/2012	60.23%
	2014/2015	55.45%
	2017	52%
Portugal [35]	2006	50.40%
	2011/2012	43.40%
	2015	50.10%
	2017	60.80%
Spain [36,37]	2009/2010	58%
	2014	56.20%
	2017	55.70%
Denmark [36]	2009/2010	48.50%
United Kingdom [36]	2011/2012	74%

## Supplementary Materials

The following are available online at https://www.mdpi.com/2076-393X/8/2/327/s1, File S1: Research data.
